# Intima-Media Thickness in Patients With Obstructive Sleep Apnea Without Comorbidities

**DOI:** 10.1007/s00408-013-9471-7

**Published:** 2013-05-14

**Authors:** Agnieszka Gorzewska, Krzysztof Specjalski, Jacek Drozdowski, Katarzyna Kunicka, Ewa Świerblewska, Leszek Bieniaszewski, Jan M. Słomiński, Ewa Jassem

**Affiliations:** 1 Department of Pneumonology, Medical University of Gdansk, ul. Debinki 7, 80-952 Gdańsk, Poland; 2 Department of Allergology, Medical University of Gdansk, ul. Debinki 7, 80-952 Gdańsk, Poland; 3 Department of Cardiology, Medical University of Gdansk, ul. Debinki 7, 80-952 Gdańsk, Poland

**Keywords:** Obstructive sleep apnea, Intima-media thickness, Cardiovascular risk factors, Ultrasonography

## Abstract

**Background:**

Obstructive sleep apnea (OSA) is associated with elevated risk of cardiovascular events. The early stages of vascular complications can be visualized by means of ultrasound. Intima-media thickness (IMT) correlates with the presence of risk factors of cardiovascular diseases such as hypertension, diabetes, tobacco smoking, or hyperlipidemia. However, little is known whether OSA itself may be the cause of IMT thickening.

**Methods:**

The study group was composed of 28 patients (6 women, 22 men; mean age = 53.8 years, mean BMI = 27.1 kg/m^2^, mean AHI = 22.4/h) with OSA who had no comorbidities. The control group consisted of 28 healthy subjects (6 women, 22 men; mean age = 53.9 years; mean BMI = 27.5 kg/m^2^). In both groups IMT was assessed in common carotid arteries with the use of ultrasonography. Additionally, in patients with OSA, pulse wave velocity, echocardiography, 24-h automated blood pressure monitoring, clinical signs and symptoms, and blood tests were performed to investigate possible correlations with IMT.

**Results:**

Median IMT was 0.41 mm in OSA patients and 0.46 mm in the control group (*p* = 0.087). Echocardiography revealed left ventricle hypertrophy in 21 %, systolic disorders in 8 %, and diastolic disorders in 57 % of the patients. In a large majority of patients, pulse wave velocity was found to be normal. IMT correlated with age (*r* = 0.446, *p* = 0.017), total cholesterol (*r* = 0.518, *p* = 0.005), daytime systolic blood pressure (*r* = 0.422, *p* = 0.025), pulse pressure 24 h and daytime (*r* = 0.424, *p* = 0.027 and *r* = 0.449, *p* = 0.019), early mitral flow/atrial mitral flow (E/A) (*r* = −0.429, *p* = 0.023), and posterior wall diameter (PWD) (*r* = 0.417, *p* = 0.270).

**Conclusion:**

In a relatively nonobese group of patients, no significant differences were found in the intima-media thickness between OSA patients without concomitant cardiovascular diseases and healthy controls. This may lead to the conclusion that IMT does not reflect increased risk of cardiovascular events in patients with isolated OSA.

## Introduction

Obstructive sleep apnea (OSA) is characterized by recurring episodes of collapse of the upper airways. At least five episodes per hour [Apnea-Hypopnea Index (AHI) > 5] and coexistence of daytime sleepiness may be found in about 4 % men and 2 % women 30–60 years of age [1]. OSA is associated with significant impairment of quality of life as well as higher mortality. He et al. [2] reported a mortality rate of 40 % in a group of patients with severe OSA during the follow-up period of 8 years. In a study by Lavie et al. [3], based on the analysis of 1,620 cases, the observed/expected mortality rate was 3.33. High mortality in OSA patients results partially from excessive daytime sleepiness which leads to car accidents and accidents at a workplace [4, 5]. Another mechanism of mortality is related to the increased risk of cardiovascular events. It has been shown that myocardial infarctions and brain strokes are two to three times more common in the population with OSA compared to healthy individuals of the same age. This is probably a consequence of arterial hypertension, endothelium dysfunction, coagulation disorders, elevated levels of inflammatory mediators, and platelet activation observed in OSA population [6].

Anatomical changes in the vessels can be visualized by means of ultrasound. However, in everyday practice, attention is usually paid to atherosclerotic plaques typical of advanced atherosclerosis with organ dysfunction. The early stages of cardiovascular disease can be evaluated by the intima-media thickness (IMT). IMT correlates with the presence of risk factors of cardiovascular diseases, such as hypertension, diabetes, tobacco smoking, and hyperlipidemia [7–9]. An IMT of ≥0.75 mm is associated with a higher risk of cardiovascular events [10].

In patients with OSA and several comorbidities, the intima-media complex is thicker than that of controls without OSA who share the same risk factors of cardiovascular events [11]. Moreover, IMT in the first group strongly correlates with frequency of desaturation below 90 % [12]. However, little is known about IMT in patients suffering from OSA alone.

The aim of this study was to investigate whether there is an increase in intima-media thickness in OSA patients without any comorbidities (arterial hypertension, diabetes, and coronary arteries disease).

## Patients and Methods

The study group was composed of 28 patients with OSA who had no comorbidities. In every case, OSA was confirmed by sleep monitoring with the use of a portable sleep recorder (Stardust, Philips Respironics, the Netherlands) that registered chest movements, air flow, saturation, heart rate, and body position. For our study we adapted the AASM criteria. Hypopnea was found when there was a 30 % reduction of airflow accompanied by 4 % oxygen desaturation or when there was a 50 % reduction of airflow accompanied by 3 % desaturation or arousal. Apnea was diagnosed when there was complete cessation of airflow [13]. All the results were visually reviewed. Sleepiness was evaluated with the Epworth scale. Patients were excluded if they had cardiovascular diseases, including arterial hypertension, coronary artery disease, diabetes, and hypercholesterolemia; a history of vascular events (myocardial infarction, transient ischemic attack, and brain stroke); current or past therapy with medications to lower blood pressure, glucose, or cholesterol level; elevated blood pressure; or abnormalities in lab tests during a visit to the clinic.

The control group of 28 subjects was recruited from the members of City Guards of Gdansk and their families. Exclusion criteria were a history of respiratory or cardiovascular disease, including diabetes, hypertension, and hypercholesterolemia; symptoms suggesting OSA; and taking medication for any of the above-mentioned conditions currently or in the past. In the control group, assessment of risk factors (medical history and laboratory tests) and IMT measurement were performed on the visit to the clinic.

### Assessment of Risk Factors

Medical history was taken and a physical examination was performed in every participant of the study. Body mass index (BMI) and waist/hip ratio (WHR) were calculated. Subsequently, blood specimens were taken from every participant for determination of glucose, HbA1c, and lipid levels. Patients with no history of cardiovascular disease or significant abnormalities in laboratory tests were included in further phases of the study.

### Intima-Media Thickness Measurement

IMT was assessed in the common carotid arteries with the use of ultrasonography (ALOKA 5000, Aloka Co., Japan) as previously described [14]. The measurement was performed bilaterally, in anterior and lateral planes, 1–3 cm below the bulb, in locations that were free of atherosclerotic plaques. Images were acquired at the end of diastole, defined as the R wave in the ECG. The images were analyzed with *cvs* ver. 1s software (Technical University of Gdansk, Poland), allowing for precise measurements in the selected fragment of the images.

Common, internal, and external carotid arteries as well as vertebral arteries were tested with Doppler ultrasound for the presence of atherosclerotic plaques and hemodynamically significant stenosis. The former was defined as local stenosis of >1.2 mm not occupying the whole circumference of a vessel and the latter was defined as a decrease of more than 50 % of the inner diameter of the vessel.

### Pulse Wave Velocity Assessment

Pulse wave velocity (PWV) was assessed in subjects lying supine after 20 min of rest. Measurements were performed simultaneously above the right common carotid artery and the right femoral artery. Pulse sensors (TY-306, Philips, the Netherlands) were placed at points where the pulse was the best palpable. Pulse wave velocity was calculated using the formula: PWV = *L*/dt, where *L* is the distance between sensors and Δ*t* (dt) is the delay between two pulse waves. A total of 20 measurements were performed for every subject. After exclusion of border values, the mean value was used for statistical analysis.

### 24-h Automated Blood Pressure Monitoring

Twenty-four-hour automated blood pressure monitoring was performed by means of oscillometry (Spacelab 90207, Montara Dolby, UK). Blood pressure was registered every 20 min during the daytime (6.00–22.00) and every 30 min during the nighttime (22.00–6.00). Mean values of systolic (SBP) and diastolic blood pressure (DBP) as well as heart rate were calculated for the 24-h period and periods representative of daytime and nighttime. According to Staessen’s criteria, only results with more than 90 % of technically acceptable parameters were analyzed [15]. As a consequence, the following data have been excluded: heart rate below 40/min or above 150/min, pulse pressure (PP; difference between systolic and diastolic pressures) lower than 10 % SBP, SBP below 50 mmHg or above 240 mmHg, and DBP below 40 mmHg or above 140 mmHg.

### Echocardiography

Echocardiography was performed with Aloka 5000 Ultrasound (Aloka Co., Japan). Luminal diameters and wall thicknesses were assessed in accordance with the Penn convention. All the parameters were measured three times and the mean value has been presented. Left ventricle dimensions were determined in the parasternal plane (M-mode). Interventricular septum diameter (IVSD), posterior wall diameter (PWD), and left ventricle diastolic diameter (LVDD) were assessed on the level of chordae tendineae of the mitral valve. Left ventricle end-systolic diameter (LVDS) was measured at the moment of the maximal front position of the heart’s posterior wall. Diastolic function of the heart was described by the following parameters: early mitral flow (E; cm/s), atrial mitral flow (A; cm/s), E/A ratio, deceleration E (DecE; cm/s^2^), and deceleration E time (DecT E; ms). Systolic function was described by ejection fraction (EF) and fractional shortening (FS). Left ventricle mass (LVM) was calculated using Devereux and Reichek’s formula [16]. Left ventricle mass index (LVMI) was defined as the quotient of LVM and body surface area.

### Statistical Analysis

The data were analyzed using the Statistica 8.0 software system (StatSoft, Tulsa, OK, USA). In order to verify the hypothesis of standard normal distribution, the Shapiro–Wilk test (W) was applied. Analysis of the relationships between normally distributed variables was based on Pearson’s correlation. In other cases, Spearman’s rank correlation was tested. The results were presented as correlation coefficient (*r*) and statistical significance (*p*). For testing independent samples, the Mann-Whitney *U* test was applied. A *p* value < 0.05 was considered significant.

The protocol of the study was approved by the Independent Ethics Committee at the Medical University of Gdansk (No. NKEBN/8/2006). All the participants gave informed consent before inclusion in the study.

## Results

The study group was composed of 28 patients with OSA, including 22 men and 6 women 29–69 years old (mean age = 53.8 years). The mean BMI was 27.1 kg/m^2^ and the WHR was 0.92. Frequency of apnea episodes was evaluated by means of polysomnography. Mean AHI was 22.4 (SD ± 11.9). Mild OSA (AHI ≤ 15/h) was diagnosed in 9 (32 %) patients, moderate OSA (AHI = 15–30/h) in 13 (46 %) patients, and severe OSA (AHI > 30/h) in 6 (22 %) patients. Sleepiness was evaluated with the Epworth scale. In 11 patients (39.3 %), the result was normal (0–9 points). Moderate sleepiness was found in 11 (39.3 %) patients, and severe daytime sleepiness was found in 6 (21.4 %) patients.

The control group comprised 28 healthy subjects: 22 men and 6 women 34–65 years old (mean age = 53.9 years). There were no statistically significant differences between the two groups in terms of age, BMI, and blood cholesterol level (Table 1).

**Table 1:** Characteristics of OSA and control groups

	OSA group	Control group	*p* value
Age (years)	29–69	34–65	
Age (mean ± SD)	53.8 ± 9.51	53.9 ± 6.2	0.434
Gender (F:M)	6:22	6:22	
BMI (kg/m^2^)	22.6–36	15.8–44.2	
BMI (mean ± SD)	27.1 ± 3.1	27.5 ± 5.1	0.737
Cholesterol level (mg/dl)	138–310	159–346	
Cholesterol level (mean ± SD)	222.2 ± 47.71	231.8 ± 36.9	0.384

Data are presented as minimal–maximal values or mean ± SD

Pulse wave velocity was determined in the study group to assess compliance of the arteries; results from 7.65 to 14.1 m/s were obtained (mean = 10.34 m/s, SD = ±1.58). In four patients, PWV exceeded 12 m/s. Statistical analysis confirmed standard normal distribution (W = 0.929, *p* = 0.06). No correlations were found between PWV and age, BMI, or AHI.

Blood pressure was measured conventionally and monitored automatically for 24 h. Mean values of systolic and diastolic blood pressure were 120.9 ± 8.86 mmHg and 78.4 ± 9.02 mmHg in conventional measurement; 121.1 ± 15.1 mmHg and 77.4 ± 8.6 mmHg in 24 h monitoring. Apart from results gained from conventional measurements, all the parameters had a normal distribution. There were no correlations between blood pressure and age, BMI, or waist circumference.

Echocardiography was performed in patients with OSA alone. Correlations were found between EF, FS, PWD and age; E, E/A, IVSD, LVM and AHI; and LVM and waist. Detailed results of 24-h blood pressure monitoring and echocardiography as well as a list of correlations are available on demand from the corresponding author.

Common carotid arteries were assessed by means of ultrasound. No abnormalities were found in 16 patients (57.2 %). Wall irregularities were found in 3 patients (10.7 %) and atheromatous plaques were revealed in 9 patients (32.1 %). They were not associated with hemodynamically significant stenosis in any case.

**Fig. 1:**
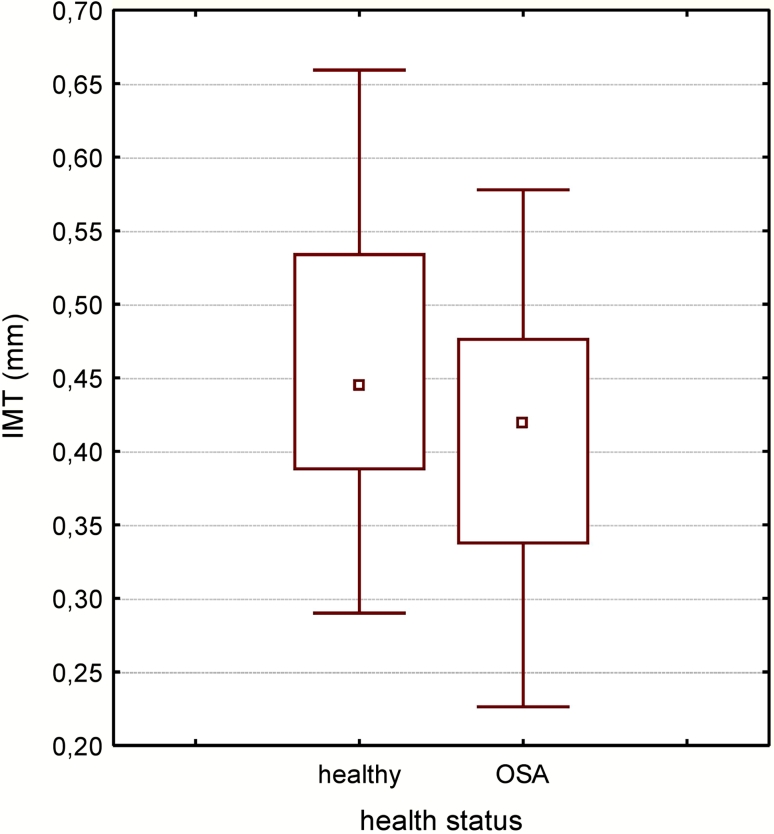
IMT in patients with OSA and in control group (*p* = 0.087). Data are presented as median (*central square*), 25–75 % (*top and bottom of boxes*), and minimal–maximal values (*top and bottom of bars*)

**Fig. 2:**
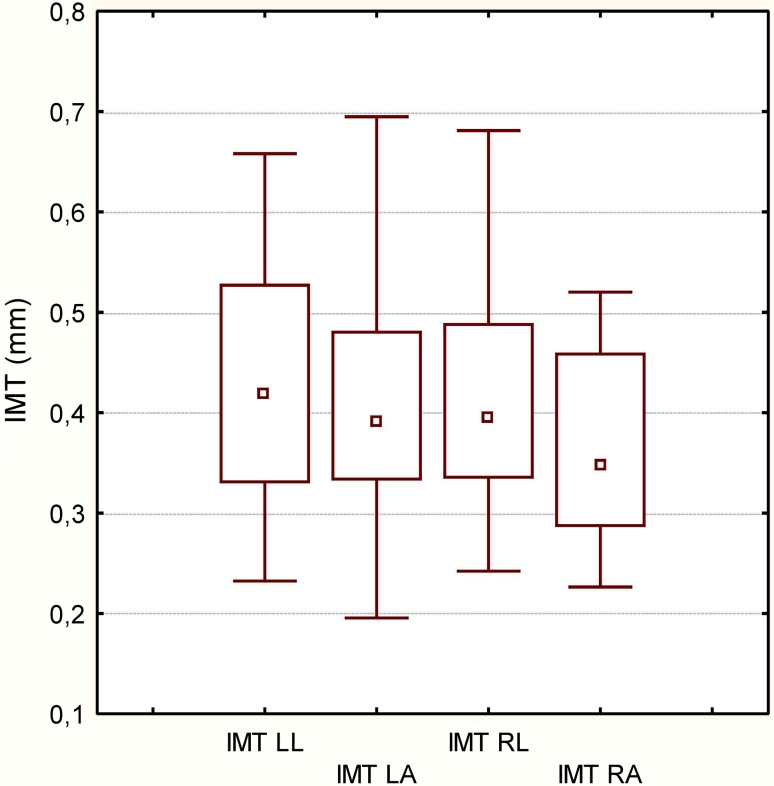
Median IMT in patients with OSA with regard to the side and plane. *LA* left common carotid artery, anterior plane, *LL* left common carotid artery, lateral plane, *RA* right common carotid artery, anterior plane, *RL* right common carotid artery, lateral plane, *NS* not significant. Data are presented as median (*central square*), 25–75 % (*top and bottom of boxes*) and minimal–maximal values (*top and bottom of bars*)

IMT was measured bilaterally in two planes: anterior and lateral. In OSA patients the highest median IMT value was registered in the left common carotid artery in the lateral plane. However, the only significant difference was observed between anterior and lateral planes in right common carotid artery (*p* < 0.05) (Fig. 1). Median IMT was 0.41 mm in OSA patients and 0.46 mm in the control group. The difference was not statistically significant (*p* = 0.087) (Fig. 2). There was no correlation between IMT and OSA severity either.

In the group of patients with OSA, we searched for correlations between mean IMT and all the anthropometric data, laboratory findings, and cardiovascular parameters. It was found that IMT correlated with age, cholesterol level, PP (24 h), PPd, SBPd, E/A, and PWD. Selected correlation coefficients (*r*) and corresponding levels of significance (*p*) are given in Table 2.

**Table 2:** Relationships between IMT and selected parameters in patients with obstructive sleep apnea

	IMT	
	*r*	*p*
Age	0.446	0.017
Cholesterol	0.518	0.005
LDL	0.641	0.001
PP 24 h	0.424	0.027
PPd	0.449	0.019
SBPd	0.422	0.025
E/A	−0.429	0.023
PWD	0.417	0.027
Desaturation <90 % time	0.128	0.17

*r* correlation coefficient, *p* level of significance

## Discussion

Obstructive sleep apnea has been shown to correlate with higher mortality resulting from cardiovascular events. Although OSA and cardiovascular diseases share many risk factors, epidemiological studies demonstrated that OSA is an independent risk factor of arterial hypertension and atherosclerosis [17]. The mechanisms of this relationship are not fully understood. They may be partly explained by constant activation of the sympathetic system, changes of pressure in the chest, and oxidative stress [18].

Atherosclerotic plaques can be visualized only at the late stages of the disease when they coexist with numerous comorbidities. Thus, in many patients with OSA, it is impossible to estimate whether the development of plaques is a consequence of recurring hypoxia only or the result of coexisting diseases. That is why assessment of the early stages of atherosclerotic changes, such as intima-media thickness measurement, seems to be a much better approach. Moreover, IMT determination could be a valuable tool in everyday practice as it is easy to determine, repetitive, and noninvasive. It is particularly indicated that IMT should be measured in patients with arterial hypertension, in whom IMT > 0.9 mm is regarded as a sign of organ dysfunction.

The main goal of the present study was to assess whether IMT is elevated in OSA patients without comorbidities (hypertension, diabetes, and coronary artery disease). When taking epidemiological data into account, it is quite clear that recruitment of patients who meet such inclusion criteria has been a difficult task. The vast majority of patients were excluded because of advanced cardiovascular diseases. Finally, 28 patients with symptomatic OSA, confirmed by portable sleep recording, were enrolled. The control group was recruited from healthy employees of the City Guards of Gdansk who did not have any chronic diseases. No significant differences were found between the groups in terms of age (53.8 vs. 53.9 years on average), gender (22 men and 6 women in both groups), and total cholesterol level (222 vs. 232 mg/dl). Mean BMI was 27.5 kg/m^2^ in the control group and 27.1 kg/m^2^ in the OSA group. In the latter group, 25 % of patients had normal BMI, in 61 % were overweight, and 14 % were diagnosed with obesity. These results are in line with previous epidemiological studies conducted in the population of northern Poland. The SOPKARD study showed the presence of overweight or obesity in 61 and 65 % of patients, respectively, in the fifth and sixth decades of life [19]. In the WOBASZ study, overweight or obesity was found in 62 % of the Polish population [20].

According to the National Cholesterol Education Program Adult Treatment Panel III (NCEP ATP III), visceral obesity is diagnosed when the waist circumference is greater than 102 cm in men and 88 cm in women [21]. This criterion was met in 6 (21 %) of our patients with OSA. The result was the same when the WHR was used to make the diagnosis.

Median IMT was 0.41 mm in patients with OSA and 0.46 mm in healthy controls (*p* = 0.087). Comparison of this result with those of previous studies is troublesome as they vary with respect to selection of participants and methodology. Most of the studies were based on typical populations of patients with OSA, i.e., they had numerous comorbidities. For example, Silvestrini et al. [11] measured IMT in 23 patients with severe, untreated OSA. The mean BMI was 31 kg/m^2^. Most of the patients had concomitant diseases such as arterial hypertension (65 %), hyperlipidemia (35 %), diabetes (17 %), and tobacco dependency (22 %). A control of similar age and risk factors (high BMI, smoking, hypertension, diabetes, and hyperlipidemia) was assigned to every patient. It was found that IMT was significantly higher in the OSA group compared to controls (1.429 ± 0.34 vs. 0.976 ± 0.17; *p* < 0.0001) [11]. Kaynak et al. [22] analyzed 114 cases who were suspected of having OSA. They were divided into three subgroups: 37 subjects with normal AHI (≤5), 41 subjects with mild-to-moderate OSA (5 < AHI < 30), and 36 subjects with severe OSA (AHI > 30). There were no significant differences among the groups in terms of age, hypertension, diabetes, smoking, and serum levels of total cholesterol and triglycerides. It was shown that IMT was higher in patients with OSA than in the group with normal AHI. The differences between the groups with mild-to-moderate and severe OSA were also significant (1.78 ± 0.57 vs. 1.91 ± 0.39, respectively; *p* < 0.001). The main limitation of that study was that the group with severe OSA (AHI > 30) was characterized by higher BMI, which correlates positively with IMT. Moreover, the patients with severe symptoms had a longer of history of OSA (8.5 years on average, compared to 7 years in the group with mild-to-moderate OSA) [22].

Both studies [11, 22] showed the relationship between OSA and thickening of intima-media complex. However, they were not sufficient to prove that OSA itself is responsible for the development of early atheromatous changes. Every risk factor, particularly diabetes, hyperlipidemia, and hypertension, might affect these relationships. For example, Savransky et al. [23] showed in an animal model that atherosclerosis developed only in mice subjected to intermittent air and fed a high-cholesterol diet. Thus, to determine whether OSA leads to an increased IMT, it was necessary to conduct studies in patients with no concomitant diseases.

Drager et al. [24] compared the IMT in a group of 30 patients with OSA (15 with mild-to-moderate OSA, i.e., mean AHI = 16.2, and 15 with severe OSA, i.e., AHI = 55.7) with that of 12 healthy controls. Patients with hypertension, diabetes, and hyperlipidemia were excluded from the study. IMT in the severe OSA group was significantly higher than that in the mild-to-moderate OSA group (0.722 vs. 0.580 mm; *p* < 0.05) and in controls (0.604 mm). However, no difference in IMT was found between the controls and patients with mild-to-moderate symptoms [24]. These relationships were also confirmed by Altin [25]. Moreover, he observed that results registered in the left carotid artery were higher in all the groups: patients with severe OSA (0.8 vs. 0.97 mm), mild-to-moderate OSA (0.63 vs. 0.78 mm), and healthy controls (0.58 vs. 0.67 mm). However, the differences were not statistically significant.

In the present study, the IMT in patients with OSA and in controls were similar. This may be because the mean AHI in our group was lower than that in the previous studies. As shown before, patients with mild and moderate OSA had an IMT comparable to that of the healthy population. There were clear differences only between patients with severe OSA and healthy subjects. We also confirmed differences between right and left common carotid arteries. It may be hypothesized that this is associated with anatomical conditions that lead to a higher blood pressure in left carotid arteries.

Significant positive correlations between IMT and age and total cholesterol were revealed. Such relationships have also been shown in previous studies [26–28]. In contrast to those studies, we have not found any correlation between IMT and BMI. This may be because the participants in our study did not have any concomitant diseases. Moreover, the mean BMI (27.1 kg/m^2^) in our study was lower than that in the studies by Drager (29 kg/m^2^), Tanriverdi (29.8 kg/m^2^), and Altin (30 kg/m^2^ in the group with severe OSA and 27.8 kg/m^2^ in the group with mild OSA) [24, 25, 29].

Although the mean IMT in our study group was not significantly elevated, atheromatous plaques were found in 32 % of patients. This is higher than that found in the studies by Tanriverdi et al. [29] and Altin et al. [25] (12.5 and 14 %, respectively). Altin et al. [25] suggested that the frequency and grade of atheromatous stenosis is higher in patients with severe OSA and hypothesized that a high AHI is an even stronger predictor of atherosclerosis than age. Tanriverdi et al. [29] did not confirm this relationship, which is in line with our findings.

Pulse wave velocity measurement was used in the study because has been demonstrated that its increase correlates with the grade of atherosclerosis [30]. It has also been found that an increase in PWV may precede development of arterial hypertension [2, 31]. So far, the relationships between PWV and OSA have been rarely investigated. Drager et al. [24] showed that PWV correlated positively with AHI (*r* = 0.61, *p* < 0.0001). PWV in cases with severe OSA (AHI > 30) was significantly higher compared to that in cases of mild-to-moderate OSA (30 > AHI > 5) and control groups. However, the difference between patients with mild-to-moderate OSA and the control group was not statistically significant [24].

In the present study, the mean PWV was normal (10.34 m/s); PWV was elevated in only four patients. No correlations have been observed between PWV and basic anthropometric parameters, IMT, or severity of OSA. This may be explained in part by the fact that the mean AHI in the study group could be classified as mild-to-moderate OSA according to Drager’s criteria. The results of previous studies on this are contradictory and make further studies necessary [32, 33].

Epidemiological studies indicate that arterial hypertension strongly depends on age and body weight. The Framingham Heart Study showed that before the age of 60, both systolic and diastolic blood pressure are increasing, while after this age SBP is still increasing and DBP is decreasing. As far as being overweight is concerned, every 10 kg above the normal body weight is associated with an increase in systolic blood pressure of about 3 mmHg [34]. This is why blood pressure was monitored in the present study, even after excluding patients with diagnosed hypertension. We found no association between anthropometric variables such as age, BMI, and waist circumference and mean blood pressure at daytime or nighttime. However, IMT correlated positively with 24-h pulse pressure (24 h PP) and daytime systolic blood pressure (SBPd). Such relationships have also been confirmed previously in populations with arterial hypertension. For example, in the ELSA study [35], SBP, PP, and age were the strongest factors that determined IMT. Similar observations are being made in normotensive patients with OSA for the first time and need to be investigated in other populations.

The structure of the left ventricle was evaluated in accordance with the European Society of Hypertension/European Society of Cardiology (ESH/ESC) guidelines [36]. The criterion of left ventricle hypertrophy was a left ventricle mass index (LVMI) of >125 g/m^2^ in men and >110 g/m^2^ in women. Six of 28 participants (21 %) met this criterion. Moreover, the PWD was elevated in two patients and the IVSD was elevated in four patients.

In epidemiological studies, left ventricle hypertrophy was found in 12–30 % of patients with arterial hypertension. It was also often found in patients with OSA. Cloward et al. [37] found left ventricle hypertrophy in 92 % of his subjects with severe OSA, defined as AHI > 40, despite the fact that only half of the group suffered from arterial hypertension. Dursunoglu et al. [38] found left ventricle hypertrophy more often in patients with severe OSA than in the groups with mild and moderate forms.

In the present study, AHI correlated with LVM and IVSD. Besides, there were positive correlations between PWD and age as well as with LVM and waist circumference. The results confirm the role of age and obesity in the pathogenesis of heart hypertrophy [39].

For assessment of the systolic function of the heart, fractional shortening and ejection fraction (EF) were used. In two patients (7 %), EF was below 55 %, and abnormalities of FS were found in five patients (17 %) (normal values were defined as 28–44 %) [40]. This is in line with that found in a study by Laaban et al. [41] who described decreased ejection fraction in 7.7 % of OSA patients. The Cardiovascular Health Study conducted in 5,201 people found that systolic function disorders occur in 1.8 % of women and in 6.3 % of men. In the population with cardiovascular diseases, the frequency of these disorders is 10.5 % [42]. Our study’s result of 7 % is only slightly higher than that observed in the general population. Ejection fraction and fractional shortening correlated with age has already been described previously [43].

In the assessment of diastolic function of the heart, early mitral flow (E), atrial mitral flow (A), E/A ratio, deceleration E (DecE), and deceleration E time (DecT E) were analyzed. In 16 (57 %) patients, there was a decrease in the E/A ratio of <1, and in 6 patients, Dec TE exceeded 240 ms. According to the guidelines of the Canadian Cardiovascular Society, all these cases could be classified as mild diastolic disorders [40]. The results are similar to those of the study by Tanriverdi et al. They evaluated mass and function of the left ventricle in OSA patients with no concomitant cardiovascular diseases and found there to be diastolic disorders in 52.5 % of participants [29]. They also found correlations between E, E/A, and AHI which were confirmed by the present study.

No significant relationships have been found between IMT and selected parameters of the systolic function of the left ventricle. However, IMT correlated negatively with E/A and positively with PWD. This is in line with the results of Parinello’s study which indicated that a higher IMT was often associated with diastolic dysfunction and heart hypertrophy [44]. This was observed in a group of 142 patients with diagnosed hypertension, which was an exclusion criterion in our study.

The main limitations of this study were its cross-sectional nature, relatively small sample size, and recruitment in a single center. As obstructive sleep apnea is usually accompanied by many comorbidities, it would be necessary to conduct multicenter studies to find a sufficient number of patients with isolated OSA.

The diagnosis of OSA was made using portable sleep monitoring, not polysomnography, which provides researchers with more data. Another limitation is that no markers of inflammation were recorded. For a better understanding of the relationships between OSA and vascular complications, monitoring of the inflammatory process seems to be necessary.

## Conclusions

In a relatively nonobese group of patients, no significant differences were found the in intima-media thickness between OSA patients without concomitant cardiovascular diseases and healthy controls. However, IMT correlated with age, total cholesterol, LDL, daytime systolic blood pressure, pulse pressure (24 h and daytime), E/A, and PWD. This may lead to the conclusion that IMT does not reflect increased risk of cardiovascular events in patients with isolated OSA.
